# Tesla Valve-Based Flexible Microhybrid Chip with Unidirectional Flow Properties

**DOI:** 10.1021/acsomega.2c02075

**Published:** 2022-08-28

**Authors:** Junyao Wang, Bowen Cui, Huan Liu, Xingyu Chen, Yunpeng Li, Rui Wang, Tianhong Lang, Hanbo Yang, lixiang Li, Hongxu Pan, Jingran Quan, Yansong Chen, Jianxin Xu, Yahao Liu

**Affiliations:** Northeast Electric Power University, Jilin 132012, China

## Abstract

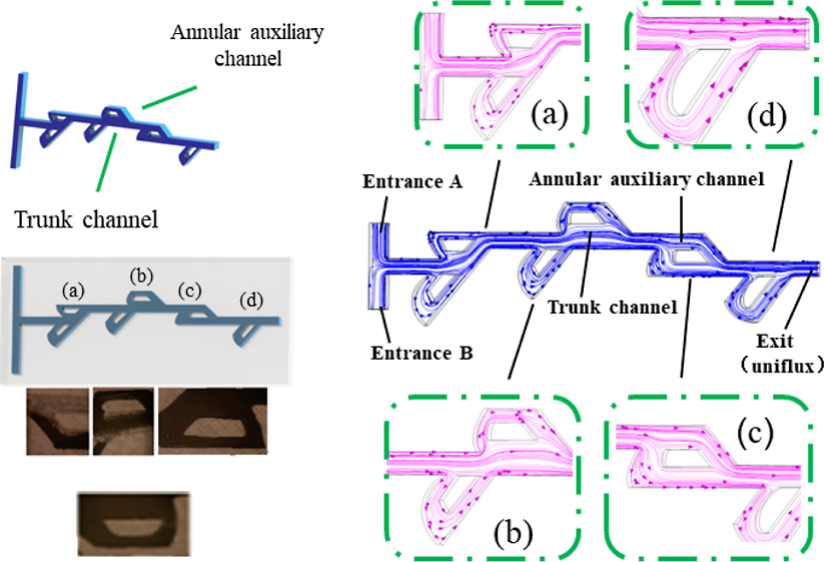

Flexible microfluidic chips have good application prospects in situations with easy bending and complex curvature. An important factor affecting the flexible microfluidic chip is its structural complexity. For example, the hybrid chip includes flow channels, mixing chambers, and one-way valves. How to achieve the same function with as few structures as possible has become an important research topic at present. In this paper, a Tesla valve micromixer with unidirectional flow characteristics is presented. A passive laminar flow Tesla valve micromixer is fabricated through 3D printing technology and limonene dissolution method. The main process is as follows: First of all, high impact polystyrene (HIPS) material was employed to make the Tesla valve channel mold. Second, the channel mold was dissolved in the limonene solvent. The mold of Tesla micromixer is made of HIPS material, the mixing experiment displace that the Tesla valve micromixer is characterized by unidirectional flow compared with the common T-shaped planar channel. At the same time, the 5-AAC Tesla valve micromixer can increase the mixing efficiency to 87%. By using four different groove structures and different flow rates of the mixing effect experiment, the conclusion is that the mixing efficiency of the 6-AAC Tesla valve micromixer is up to 0.89 when the flow rate is 2 mL/min. The results manifest that the Tesla valve structure can effectively improve the mixing efficiency.

## 1 Introduction

As an important part of microsystems, micromixers are mainly used to realize efficient mixing of two or more different reactants in the microscale space.^1,9^ Currently, common micromixers maintain one-way flow by attaching an energy field to the outside of the flow channel.^17^

In recent years, microfluidic drive by micro–nano devices such as micropump and microvalve has attracted wide attention; Amirhesam et al. studied a pneumatic peristaltic micropump.^4^ The thermoplastic polyurethane elastomer rubber elastic film is deformed, and the fluid channel below is squeezed. The three actuators work in a specific sequence to realize one-way transportation of the fluid.

In order to enable the micromixer to be stably controlled according to a specific time series,^20^ Feng et al. developed a cam linear peristaltic micropump.^5^ Soft lithography was used to fabricate flexible microfluidic pipes,^18^ and 3D printing technology was used to fabricate a microcam follower system. The microfluidic channel is compressed synchronously by three microcam follower systems with different phase angles relative to the camshaft, and the pump has the advantages of simple structure and easy to control.

Jang et al. studied valveless electromagnetic travelling wave micropumps composed of polymethyl methacrylate, PDMS, and other materials.^7^ The pump drives the micropipe fluid through a permanent magnet and moves in a travelling wave manner and has the advantages of fast response and high efficiency.

The peristaltic micropump proposed by Smits is fabricated using a MEMS process. This micropump has three piezoelectric actuators,^16^ which are energized in sequence, and the six-step sequence is used for peristaltic transmission with a maximum flow rate of 3 μL/min.

Yu et al. designed a piezoelectric-driven peristaltic micropump.^19^ The system consists of a 12 V power supply, a microprocessor, a differential amplifier, a phase controller, an analog-to-digital converter, and so forth. By studying the dynamic behavior of the diaphragm and comparatively analyzing the influence of different phase signal changes on the performance, the results show that the maximum reverse pressure can reach 520 Pa.

ChanJeong proposed a thermoelectric peristaltic micropump.^8^ The diameter of the actuator diaphragm is 205 mm, and the thickness is 30 μm. When the input voltage is 20 V and the driving frequency is 2 Hz, the maximum flow rate of the micropump is about 0.36 μL/s.

Although these micropumps have the characteristics of rapid response and unidirectional flow of reagents, the fluid inside the micropump is exposed to the electric field, magnetic field, sound field, and thermal field. Experimental results have an impact. At the same time, the manufacturing process of the micropump is complicated and the cost is high, the integrated micropump is not easy to clean, and there are risks such as cross-contamination. In view of the above problems, combined with the 3D printing technology and the polymer dissolution technology, this paper proposes a new Tesla valve structure micromixer. A micromixer with a one-way flow characteristic of the Tesla valve channel is produced without keys and other processing steps. At the same time, the hybrid efficiency curve is obtained through the simulation of three kinds of structures. Furthermore, the unidirectional flow of the micromixer and the influence of different flow rate groove structures on the mixing efficiency are verified by experiments. Finally, a unidirectional flow-through Tesla valve micromixer with high efficiency was fabricated.

## 2 Preparation and Experiment

**Figure 1:**
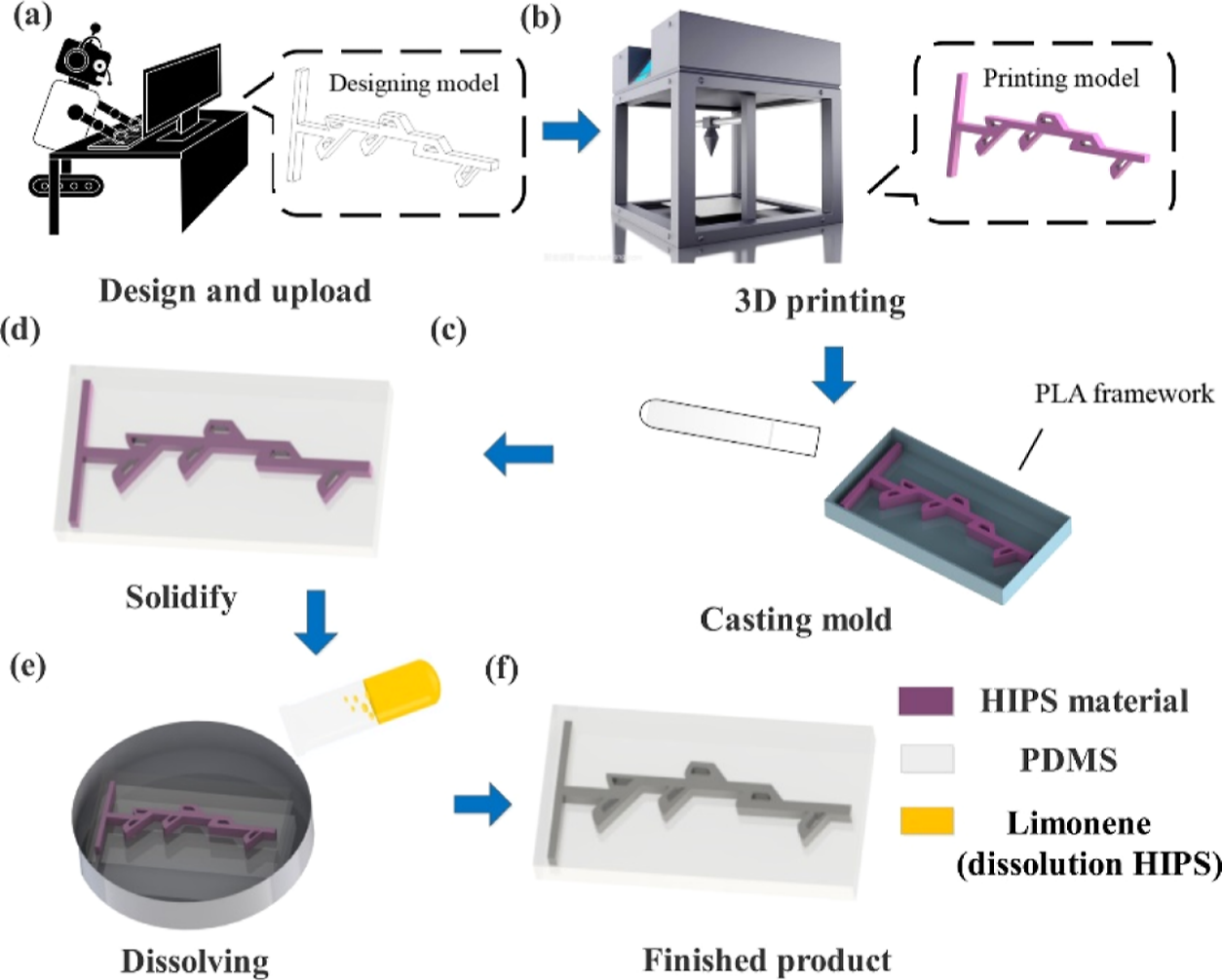
Schematic diagram of the Tesla valve micromixer. (a,b) Printing the HIPS mold. (c) Printing the PLA framework and casting. (d) Punching the holes. © Dissolving the microchannel mold in limonene solvent. (f) Completing the chip.

Figure 1 demonstrates the fabrication principle of the Tesla valve structure micromixer. The micromixer is fabricated by 3D printing technology. It is worth noting that the channel of the mixer is achieved by dissolving the HIPS mold material through limonene. The specific production process and data are shown in Table 1.^11^

**Table 1:** Manufacturing Steps and Data of 3D Tesla Valve Micromixer

steps	the production process	specific methods and parameters
step (a) and (b)	printing the HIPS mold	using 3D printing and HIPS to print
		Tesla valve molds
step (c)	printing the PLA framework and casting	3D printing technology is employed to fabricate the PLA framework with the size of 5 × 2 × 1.5 cm. Then PDMS is poured into the molds. Heat curing is carried out with the temperature of 80° and the storage time is 20 min
step (d)	punching the holes	the chip is perforated with a 2 mm diameter hole punch
step (e)	dissolving the microchannel mold in limonene solvent	limonene (the concentration of 99%) is used to dissolve the channel and the dissolution time is 30 min
step (f)	completing the chip	a micro mixer with the Tesla valve micro-channel is fabricated.

Sylgard 184 silicone elastomer and curing agent were purchased from Dongguan Sanbang New Material Technology Company in China. The model of the 3D printer was RAISE 3D Pro2 plus. Polylactic acid (PLA) and HIPS were purchased from Flashforge, China. The fluid was simulated by COMSOL simulation software. Limonene solution was purchased from Guangzhou Prek Chemical company. Flow rate was controlled by a jet pump (LSP02-2B, Longerpump) for the mixing experiment. The mixing effect was observed by an inverted fluorescence microscope (OLYMPUS IX73, Japan).

The experiment uses a perfusion syringe pump (LSP02-2B, Longerpump) to achieve liquid filling and delivery. It is controlled by a computer and calibrated automatically by the syringe pump system, and the flow rate of the micromixer structure is increased by increasing the linear speed and the cross-sectional area of the T-shaped inlet of the micromixer per unit time. The parameters of the syringe pump are as follows: flow range, 0.001 μL/min–43.349 mL/min; maximum stroke, 140 mm; syringe linear speed range, 5 μm/min–65 mm/min; stroke control accuracy, error ≤ ±0.5%.

The experimental steps for making the micromixer are shown in Table 1. In step 1, the micromixer mold is modeled by 3D drawing software and printed using a 3D printer and HIPS material; step 2 describes the use of 3D printing technology to make a PLA frame with a size of 5 × 2 × 1.5 cm. In step 3, the silicone elastomer PDMS and curing agent are mixed in a ratio of 10:1, and then half of them is poured into the PLA frame and heated for 20 min. In step 4, the HIPS micromixer mold is placed on the solidified PDMS; then the other half is poured into the frame, and the HIPS mold is covered and put into a drying box to solidify. Step 5 explains the drilling of a solidified mold in the PLA framework; in step 6, limonene is poured into the hole, and the chip preparation is completed in 30 min.

## 3 Simulation Analysis

### 3.1 Fluid Flow Characteristics and Efficiency Advantages of Tesla Structures

**Figure 2:**
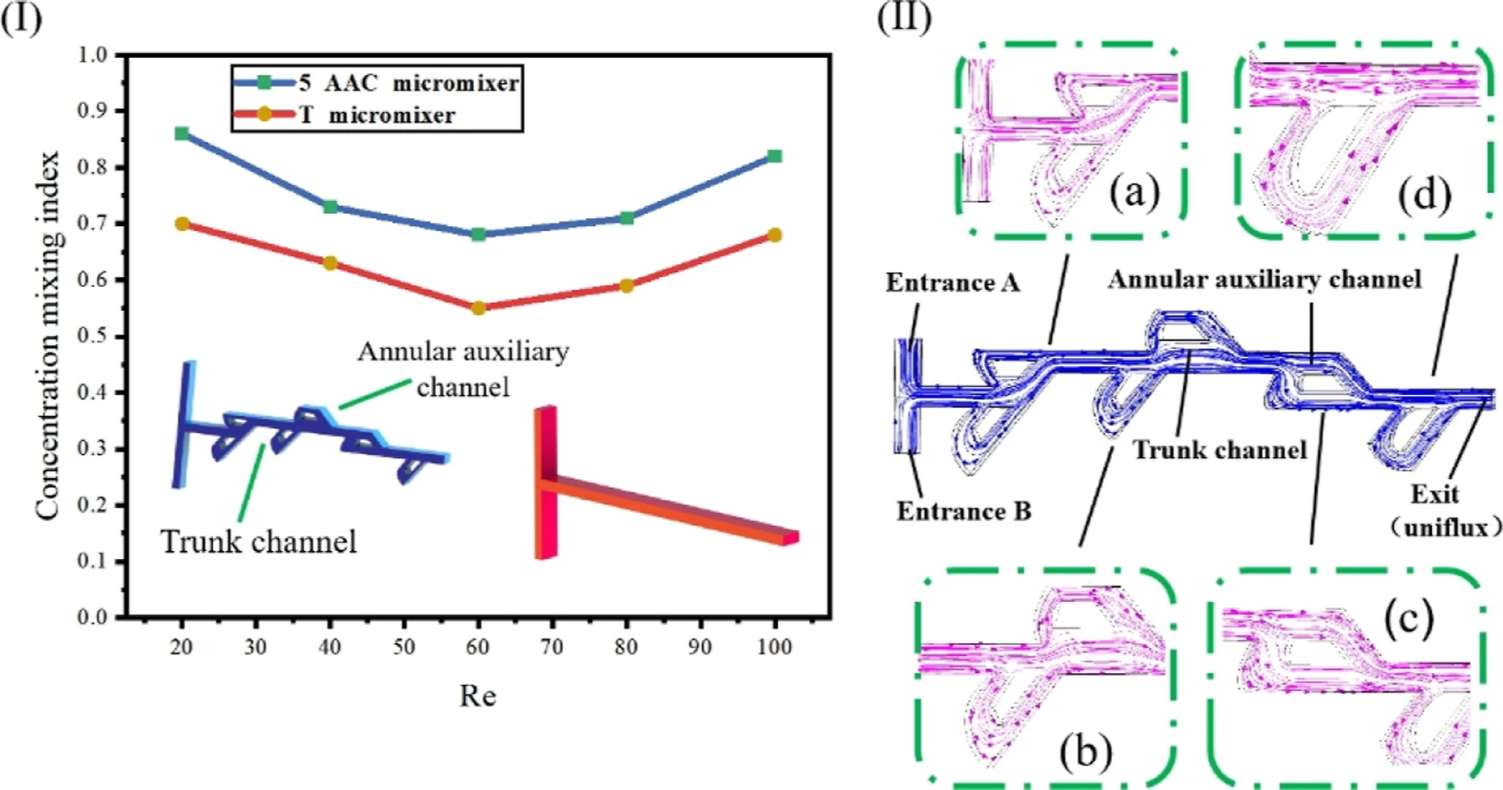
(I) Comparison of mixing efficiency between the T-type structure and 5-AAC (annular auxiliary channel and trunk channel) Tesla valve structure at different Reynolds numbers; (II) ionic diffusion mixing model of the Tesla valve structure micromixer. (a––d) are partial enlarged views of the ion diffusion model of the annular auxiliary channel and the main channel.

Figure 2I demonstrates the comparison of simulation efficiency between the T-channel and Tesla valve micromixer.^3,5,10^ When *Re* is between 20 and 60, the mixing efficiency of the Tesla valve micromixer gradually decreases with the increase of Reynolds number. When *Re* is between 60 and 100, with the increase of Reynolds number, the mixing efficiency of the Tesla valve micromixer gradually increases. When *Re* is between 20 and 60, the mixer is not easy to work due to the large viscous force and small inertia force of the fluid. At this time, the mixing efficiency decreases with the increase of *Re*. When *Re* is between 60 and 100, the mixing speed of the two fluids accelerates obviously with the increase of the inertia force. The increased mixing speed increases the inertia effect of the fluid, so the mixing time of the microchannel is shortened and the mixing efficiency is accelerated. Figure 2II shows the mixing model of ions in the diffusion process. In Figure 2II, since the mixing area of the main channel of the Tesla valve micromixer is relatively flat, the movement intensity of the fluid in the channel is small and the lateral diffusion of the molecules is not severe, which ultimately makes the mixing effect of the fluid worse. When the fluid is in the annular auxiliary channel, the velocity and force of the fluid at the bend varies greatly. According to eqs 1 and 2, the change of velocity will increase the momentum of motion, increase the degree of convection, and increase the friction force generated by viscosity, thereby strengthening the shearing effect of the fluid and improving the mixing efficiency.
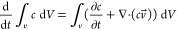
1
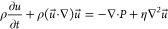
2 is the frictional force due to viscosity; −∇·*P* is the force due to the pressure gradient; *F⃗* is the external force acting on the fluid; *P* is the internal pressure of the fluid; *u⃗* is the fluid flow vector velocity.

When *Re* is the same, the maximum mixing efficiency difference between the Tesla valve channel and T-channel is close to 16%. The calculation method of mixing efficiency is as follows^3^
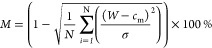
3where *N* is the number of selected points; *W* is the fluid mass fraction corresponding to the node on the monitoring surface; *c*_m_ is the fluid mass fraction corresponding to the point of full fluid mixing on the monitoring surface; σ is the deviation of the mass fraction of the fluid on the monitoring surface without mixing. *M* is the mixed intensity, and the value is 0–1. The Tesla valve micromixer has a complex channel shape. When the two liquids flow in the channel, the polygonal groove will increase the molecular diffusion, resulting in a more drastic change in the liquid flow. Compared with the planar T-type structure, the Tesla valve micro mixer has a stronger mixing performance.

### 3.2 Research on the Tesla Channel Structure and the Mixing Efficiency

In order to study the relationship between the fluid mixing efficiency and the structure in the Tesla valve channel, first, three 3D geometric models of Tesla valve with different number of grooves were drawn with SolidWorks, and then numerical simulation was imported. The simulation divided the calculation area into grids to carry out network division of the Tesla valve structure. The specific table data is shown in Table 2.

**Table 2:** Numerical Model Detailed Information

property	value
mesh vertices	154,674
number of elements	201,414
minimum element quality	0.0383
average element quality	0.65
element volume ratio	5.446 × 10^–4^
mesh volume (mm3)	75.4

**Figure 3:**
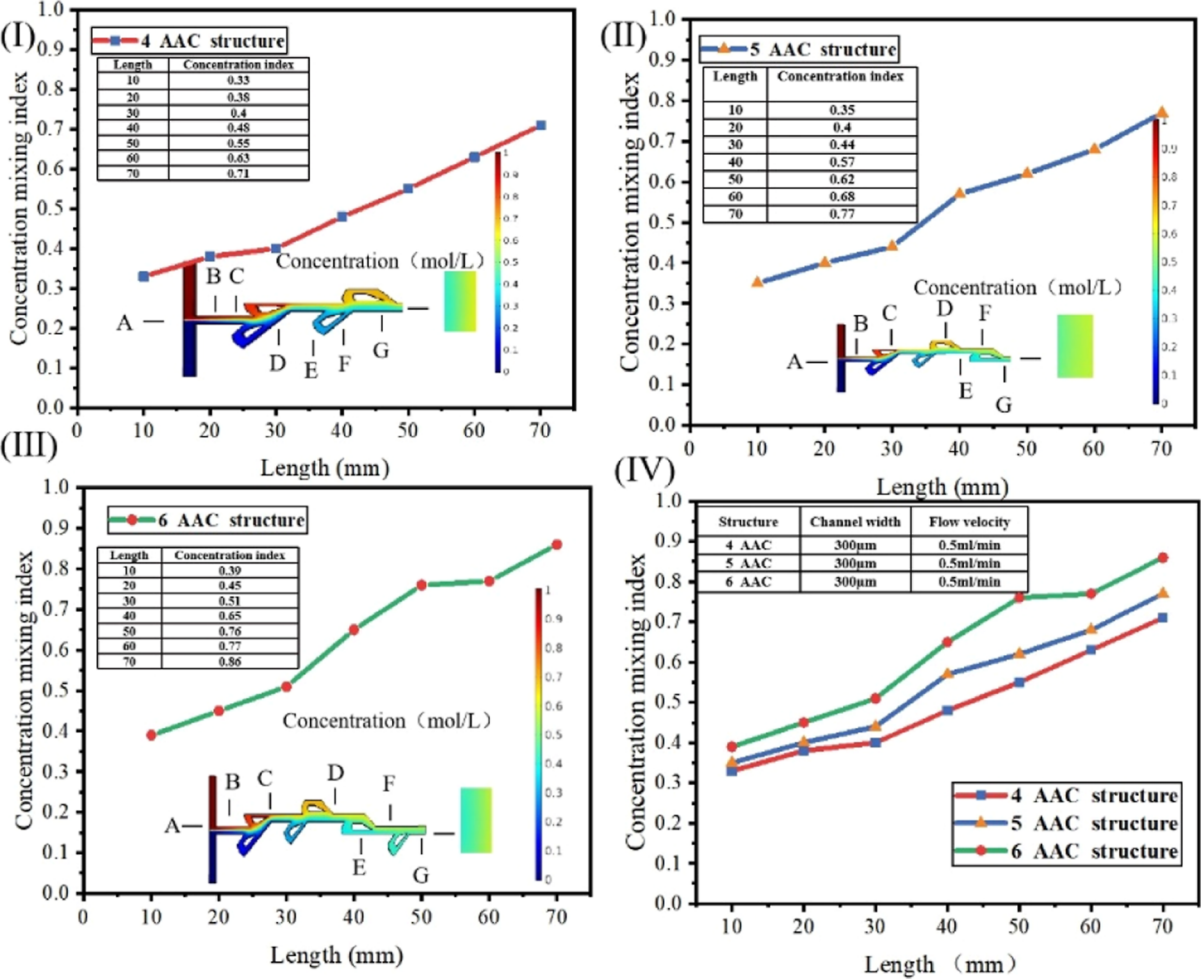
Hybrid simulation diagram of the Tesla valve structure micromixer at 0.5 mL/min. A–G represents the end point of the micromixer at 10–70 mm nodes. The color legend represents the solution concentration. (I) Mixing efficiency value of the micromixer in a 4-AAC Tesla valve structure. (II) Mixing efficiency value of the micromixer in a 5-AAC Tesla valve structure. (III) Mixing efficiency value of the micromixer in a 6-AAC Tesla valve structure. (IV) Mixing efficiency value of the micromixer in 4-, 5-, and 6-AAC Tesla valve structures.

Figure 3 shows the mixing simulation data under different grooves at a flow rate of 0.5 mL/min. Figure 3I shows the simulated mixing efficiency value of the micromixer with a length of 10–70 mm in a 4-AAC Tesla valve structure at a flow rate of 0.5 mL/min. As the size of the micromixer gets longer and longer, the mixing efficiency increases. This is because the larger the size of the micro mixer, the longer the mixing time of the fluid in the channel, which increases the contact time of the fluid and improves the mixing efficiency. Figure 3II,III simulates the mixing efficiency values of 5- and 6-AAC structures at 0.5 mL/min flow rate. As the number of AAC increases, the two high-concentration fluids cross and stretch at the boundary of the micromixer, thus increasing the contact area between each other, enhancing the mixing of fluids, and leading to the increase of mixing efficiency. Figure 3IV shows the comparison diagram of simulation trends of the three AAC structures. Finally, it is concluded that the mixing efficiency of the 70 mm micromixer with 6-AAC structures is 87% when the flow rate is 0.5 mL/min.

**Figure 4:**
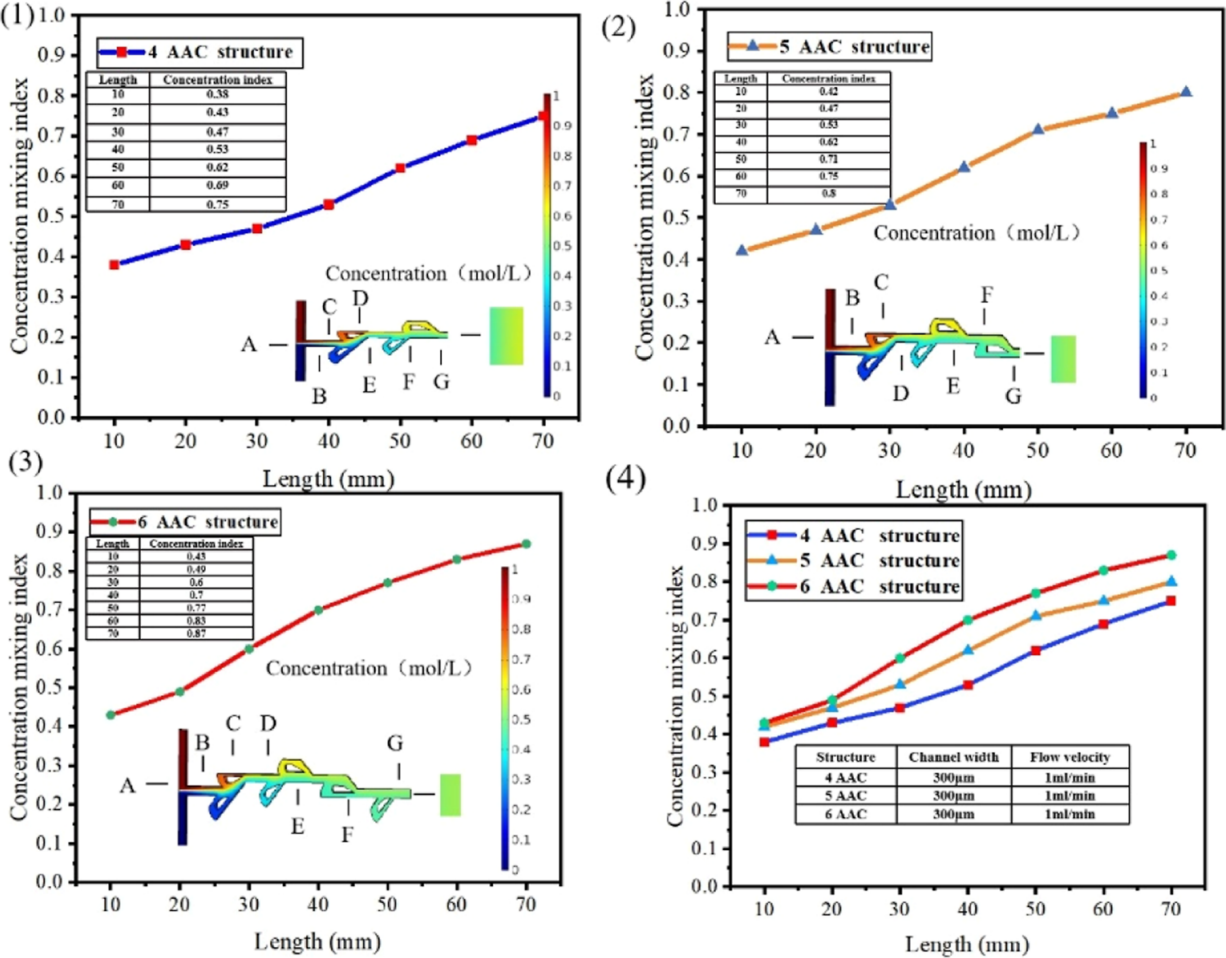
Hybrid simulation diagram of the Tesla valve structure micromixer at 1 mL/min. A–G represents the end point of the micromixer at 10–70 mm nodes, respectively. The color legend represents the solution concentration. (I) Mixing efficiency value of the micromixer in a 4-AAC Tesla valve structur. (II) Mixing efficiency value of the micromixer in a 5-AAC Tesla valve structure. (III) Mixing efficiency value of the micromixer in a 6-AAC Tesla valve structure. (IV) Mixing efficiency value of the micromixer in 4-, 5-, and 6-AAC Tesla valve structures.

Figure 4 shows the mixing simulation data under different grooves at a flow rate of 1 mL/min. Figure 4I shows the simulated mixing efficiency value of the micromixer with a length of 10–70 mm in a 4-AAC Tesla valve structure at a flow rate of 1 mL/min. Figure 4II,III simulates the mixing efficiency values of 5- and 6-AAC structures at 1 mL/min flow rate. When *Re* is between 20 and 60, as the Reynolds number increases, the mixing efficiency of the Tesla valve micromixer gradually decreases. When *Re* is between 60 and 100, the mixing efficiency of the Tesla valve micromixer gradually increases with the increase of Reynolds number. When *Re* is between 20 and 60, due to the large viscous force of the fluid and the small inertial force, the increase of the Reynolds number at this time will not increase the mixing efficiency.^21^ When *Re* is between 60 and 100, with the increase of the Reynolds number, it can be seen from formula 4 that the Reynolds number is proportional to the flow. Other things being equal, the Reynolds number increases with the flow. When the Reynolds number is large, chaotic fluid is produced. Mixing efficiency does not increase monotonically with the flow rate but increases with the flow rate when the fluid density, channel characteristic size, and dynamic viscosity are constant. As the flow rate increases, the mixing efficiency increases. Because when the flow rate increases, the mixing state dependent on molecular diffusion is broken, chaotic convection occurs in the groove channel to a certain extent, and the mixing efficiency is improved.
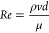
4where ρ is the density of the fluid, *v* is the characteristic velocity of the fluid, *d* is the characteristic dimension of the channel, and *v* is the dynamic viscosity of the fluid. Figure C compares the mixing efficiency of the T-shaped configuration with the 5-AAC (annular auxiliary channel and main channel) Tesla valve configuration at different Reynolds numbers.

The laminar flow model and the thin layer mass transfer model were used for numerical simulation. In the simulation, the wall of the microchannel is set as a nonslip boundary condition, and no pressure is set at the outlet. The flow field is obtained by Navier-Stokes^2,14^ equations, and the fluid mixing is obtained by convection-diffusion equations, as shown in eqns 5 and 6
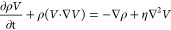
5
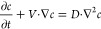
6

ρ is the fluid density, η is the hydrodynamic viscosity, *P* is the fluid pressure, *V* is the velocity vector, *C* is the concentration, and *D* is the intermolecular diffusion coefficient. The Tesla valve structure can achieve the maximum mixing efficiency of 0.885. The numerical definition parameters and boundary condition settings involved in simulation are shown in Tables 3 and 4.

**Table 3:** Numerical Simulation of Boundary Conditions

	entry 1	entry 2	exit	wall surface
the flow field	*P* = 0	*P* = 0	*P* = 0	*u* = 0
ion concentration field	*c*1 = 1 mol/m^3^	*c*_2_ = 2 mol/m^3^		*n* = 0

**Table 4:** Numerical Simulation of Variable Parameters

the serial number	cross-sectional area of the channel	number of grooves	viscosity coefficient
1	200 × 200 μm	4	8.55 × 10^–4^
2		5	
3		6	

## 4 Results and Discussion

### 4.1 Tesla Channel Unidirectional Flow Experiment

**Figure 5:**
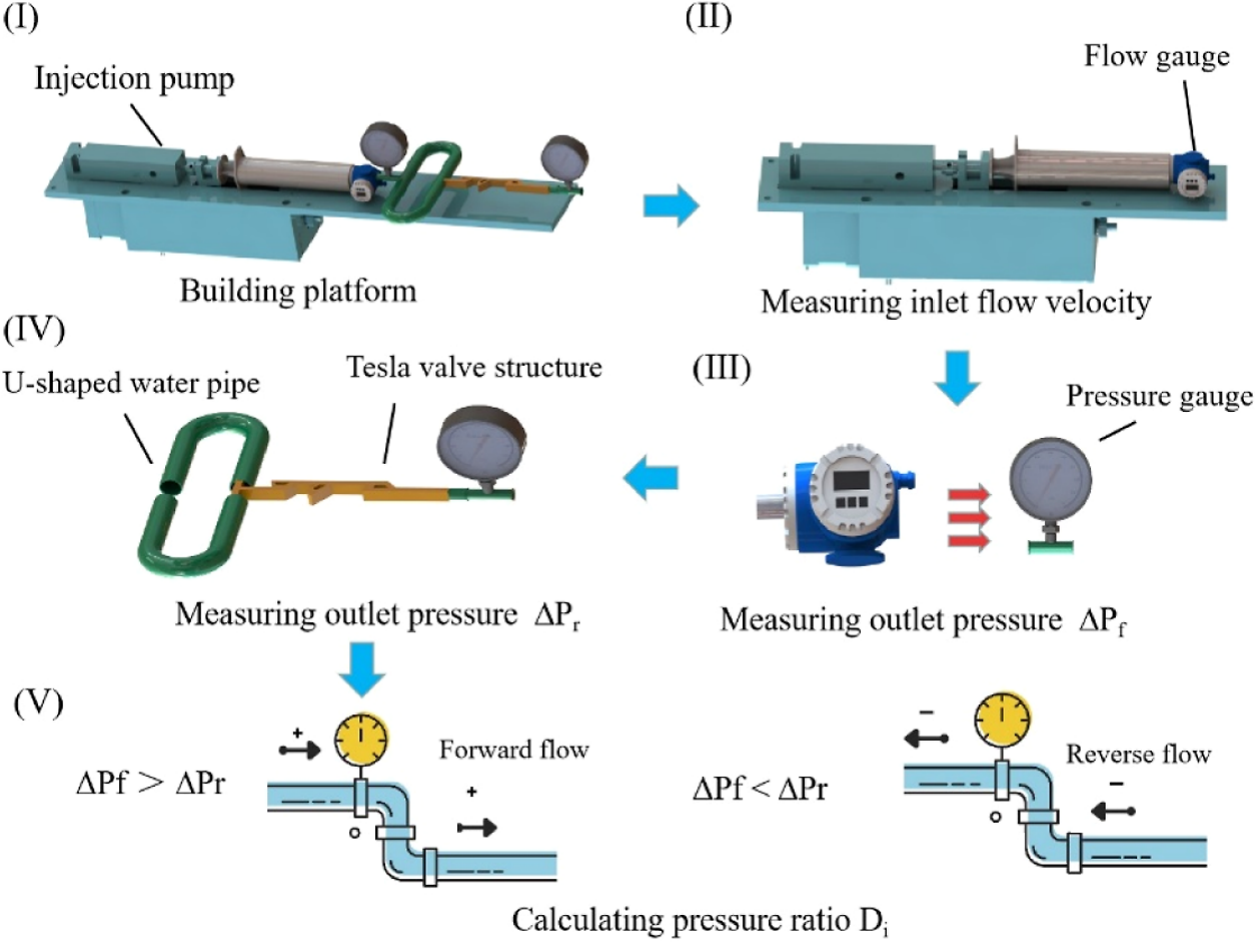
Unidirectional flow test procedure.

**Figure 6:**
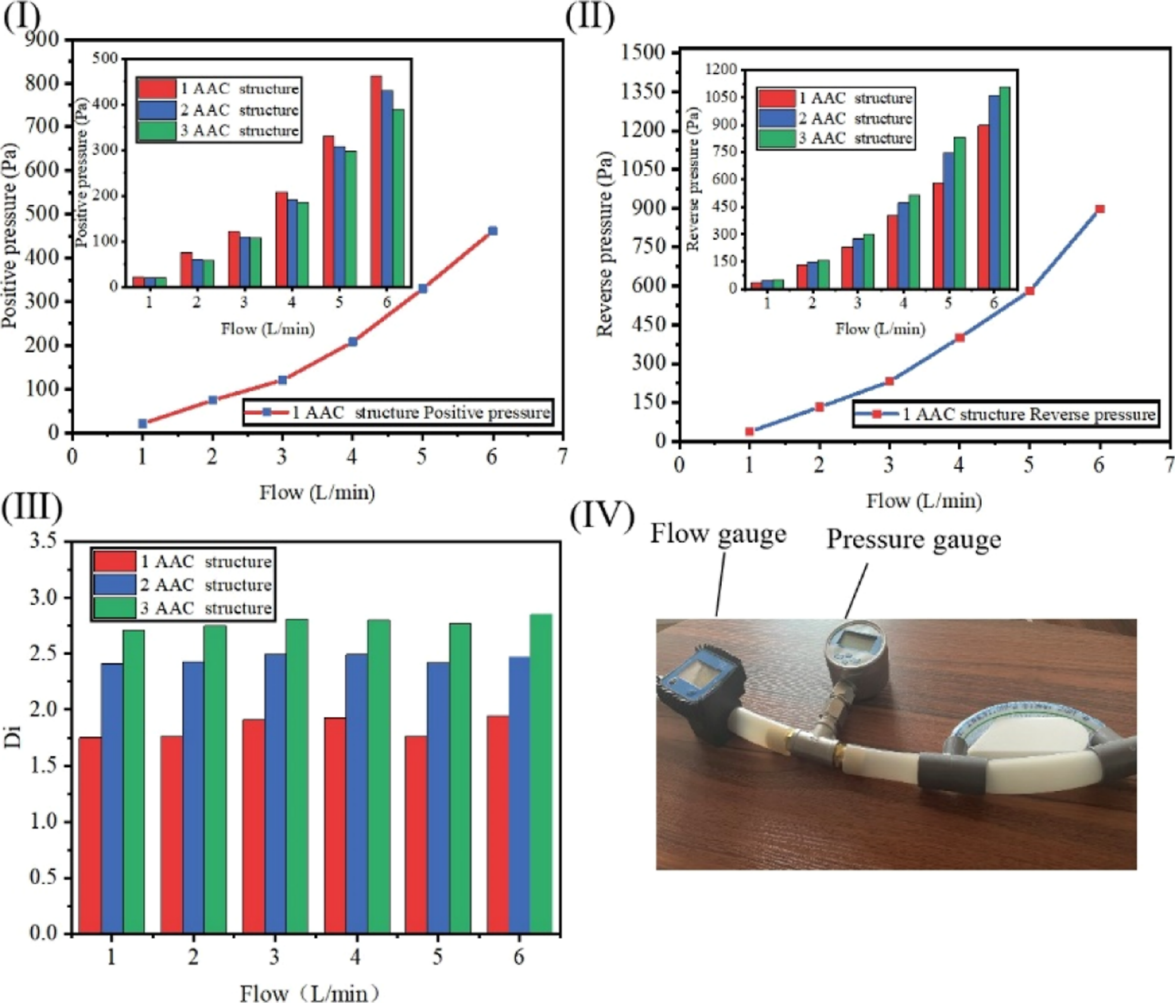
Effect of different flow rates on the pressure and the experimental apparatus diagram. (I) Forward pressure at different flows. (II) Reverse pressure at different flows. (III) *D*_i_ at different flow rates and configurations. (IV) Experimental setup.

Before the mixing efficiency experiment, first the Tesla valve structure is verified for the characteristics of single flow. Figure 5 and Table 5 present the production process of a single flow experiment. Figure 5 shows the influence of different flows on pressure and the diagram of experimental devices. Figure 6I is the forward pressure at different flows, and Figure 6II is the reverse pressure at different flows. It can be seen that the pressure increases with the increase of flow. As the pipeline resistance is proportional to the square of the flow, the flow accelerates, the pipeline resistance increases, and the pressure increases. Figure 6III is the ratio of forward pressure to reverse pressure at different flows. The equation of pressure ratio is shown as^7^
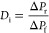
7

**Table 5:** Unidirectional Flow Test Procedure

steps	the experimental process	specific methods and steps
step 1	building experimental platform	the equipment is formed from (composed of) water faucet, flow gauge, pressure gauge, and Tesla valve structure mold
step 2	measuring inlet flow velocity	flow velocity is measured by flow gauge
step 3	measuring inlet pressure Δ*P*_f_	inlet pressure is measured by pressure gauge at different flow velocity (rates). The pressure gauge is placed on the left of the model
step 4	measuring outlet pressure Δ*P*_r_	outlet pressure is measured by pressure gauge at different flow velocities. The pressure gauge is placed on the right of the model
step 5	calculating pressure ratio *D*_i_	calculating the ratio of the positive pressure to reverse pressure. When Δ*P*_f_ > Δ*P*_r_, it is proved that the device has good forward flow. When Δ*P*_f_ < Δ*P*_r_, the device has poor forward flow
step 6	following the above method to measure other data and calculating	calculating the pressure ratios of the other two structures as described above

*D*_i_ represents the pressure ratio, Δ*P*_r_ is the pressure generated by the channel in reverse flow, and Δ*P*_f_ is the pressure generated by the channel in forward flow. The larger the *D*_i_ is, the more difficult the reverse flow is than the forward flow, and the more obvious the effect of one-way flow is. The pressure ratio increases with the increase of the groove structure. This is because the circular channel and the horizontal channel of the groove structure have large geometric mutations. When the fluid flows in the reverse direction to the groove position, the resistance is increased, so the reverse pressure is much greater than the forward pressure. At the same time, the increase of the number of grooves will have a superposition effect on the increase of the pressure ratio. Finally, the pressure ratio of the 3-AAC Tesla valve structure is the maximum, which is 2.75, and the one-way flow of the device is the best. Figure 6IV is the experimental device diagram, which consists of a water faucet, a flow gauge, a Tesla valve structure mold, a water pipe, and connectors. The experiment is verified by controlling the flow rate and measuring the pressure at the inlet and outlet and calculating the pressure ratio. The experiment of Figure 6 is to put the micromixer inside the rubber tube, measure the pressure expression, and calculate the *D*_i_ value by controlling the flow rate. The parameters of the rubber tube and the micromixer are given in the text.

Micromixer parameters are as follows: length 70 μm, width 15 μm, and height 10 μm.

Rubber tube parameters are as follows: inner diameter 5 mm; outer diameter 7 mm; fixed on the flow meter and pressure gauge through a circular distributor and adapter.

### 4.2 Effect of Different Grooves on Mixing Efficiency

**Figure 7:**
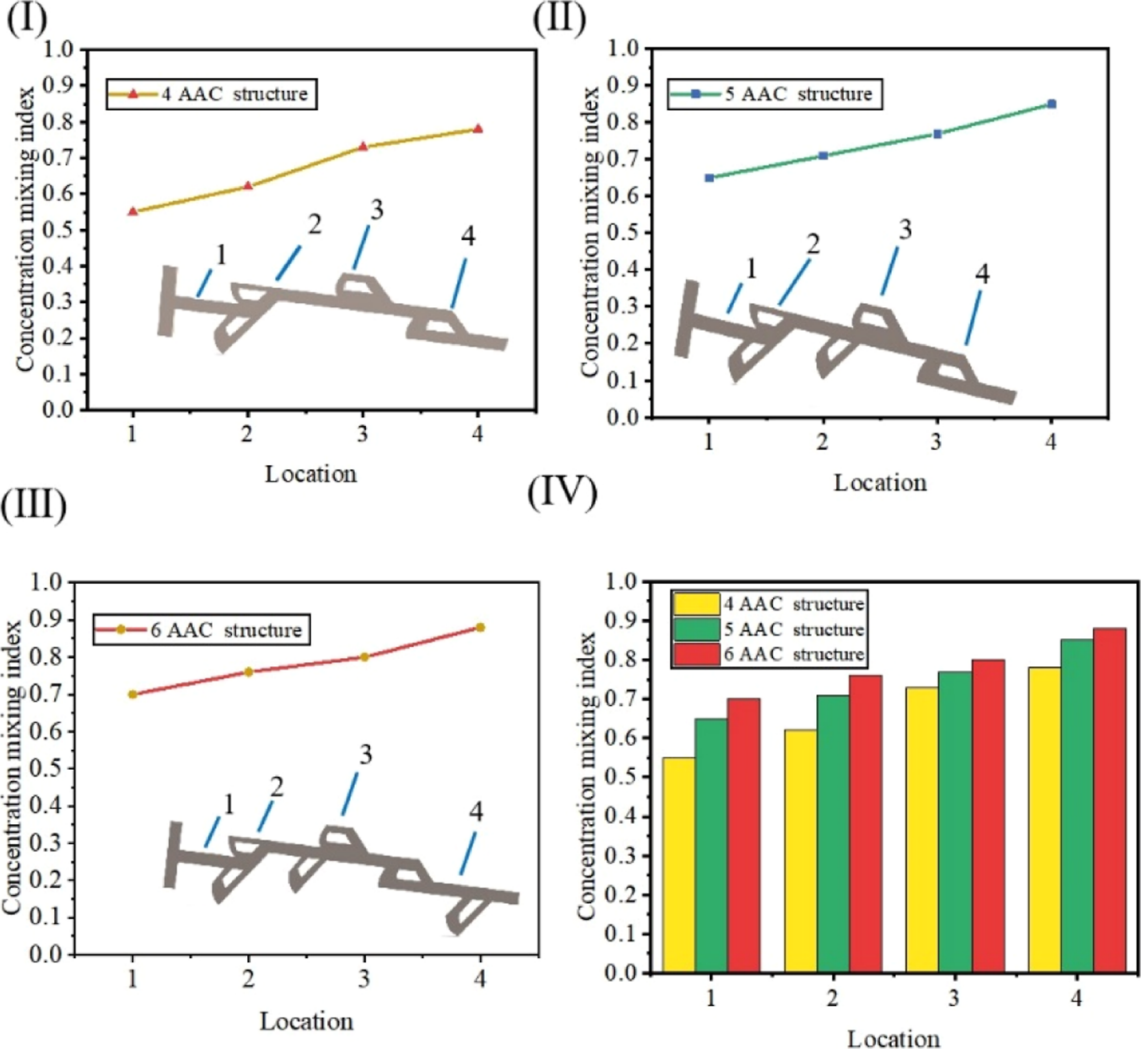
Effect of different grooves on the mixing efficiency. (I) Mixing efficiency of the 4-AAC Tesla valve micromixer at different positions. (II) Mixing efficiency of the 5-AAC Tesla valve micromixer at different positions. (III) Mixing efficiency of the 6-AAC Tesla valve micromixer at different positions. (IV) Mixing efficiency of the 4-, 5-, and 6-AAC Tesla valve micromixers at different positions.

Figure 7 demonstrates the mixing efficiency curves of micromixers with the Tesla valve structure and three different groove numbers. 1, 2, 3, and 4 are the measurement positions of the corresponding 3D schematic diagram of the three structures’ mixing efficiency. Figure 7I shows the mixing efficiency of the 4-AAC Tesla valve micromixer at different positions. With the increase of flow time, the mixing efficiency increases from 0.55 to 0.78. The longer the fluid stays in the microchannel, the higher the mixing efficiency. Figure 7II,III shows the mixing efficiency of 5-AAC and 6-AAC Tesla valve micromixers at different positions. It shows that the mixing efficiency increases with the increase in the number of grooves. This is because when the fluid passes through the groove of the micromixer, part of the shunt will leave the original flow direction and flow to the direction of the protruding object surface. The typical flow characteristic of microfluidics is laminar flow. The fluid layers are in contact with each other and do not react. At this time, the free diffusion between fluid molecules is the dominant factor of mixing. Equation 8 shows the diffusion time *t* of molecules is proportional to the square of the diffusion distance *d*^2^ and inversely proportional to the diffusion coefficient *D* of the medium, that is to say, reducing the diffusion distance *d* between molecules and increasing the diffusion coefficient *D* of the fluid are both the same. It is beneficial to improve the efficiency of microfluidic flow. At the same time, the increase of the flow rate destroys the immiscible equilibrium flow state between the fluid layers during the microfluidic flow process. The splitting and reorganization are improved, thereby improving the mixing efficiency. Figure 7IV shows the trend comparison of mixing efficiency of three structures. Figure 7IV shows the channel parameter comparison of the micromixer. Obviously, when the micromixer height is 5 mm, the channel width is 200 μm, and the cross-sectional area is 40,000 μm^2^, the 6-AAC Tesla valve micromixer has the highest mixing efficiency of 0.88.
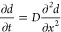
8where *d* is the diffusion distance and *D* is the diffusion coefficient.

### 4.3 Effect of Different Flow Rates on Mixing Efficiency

**Figure 8:**
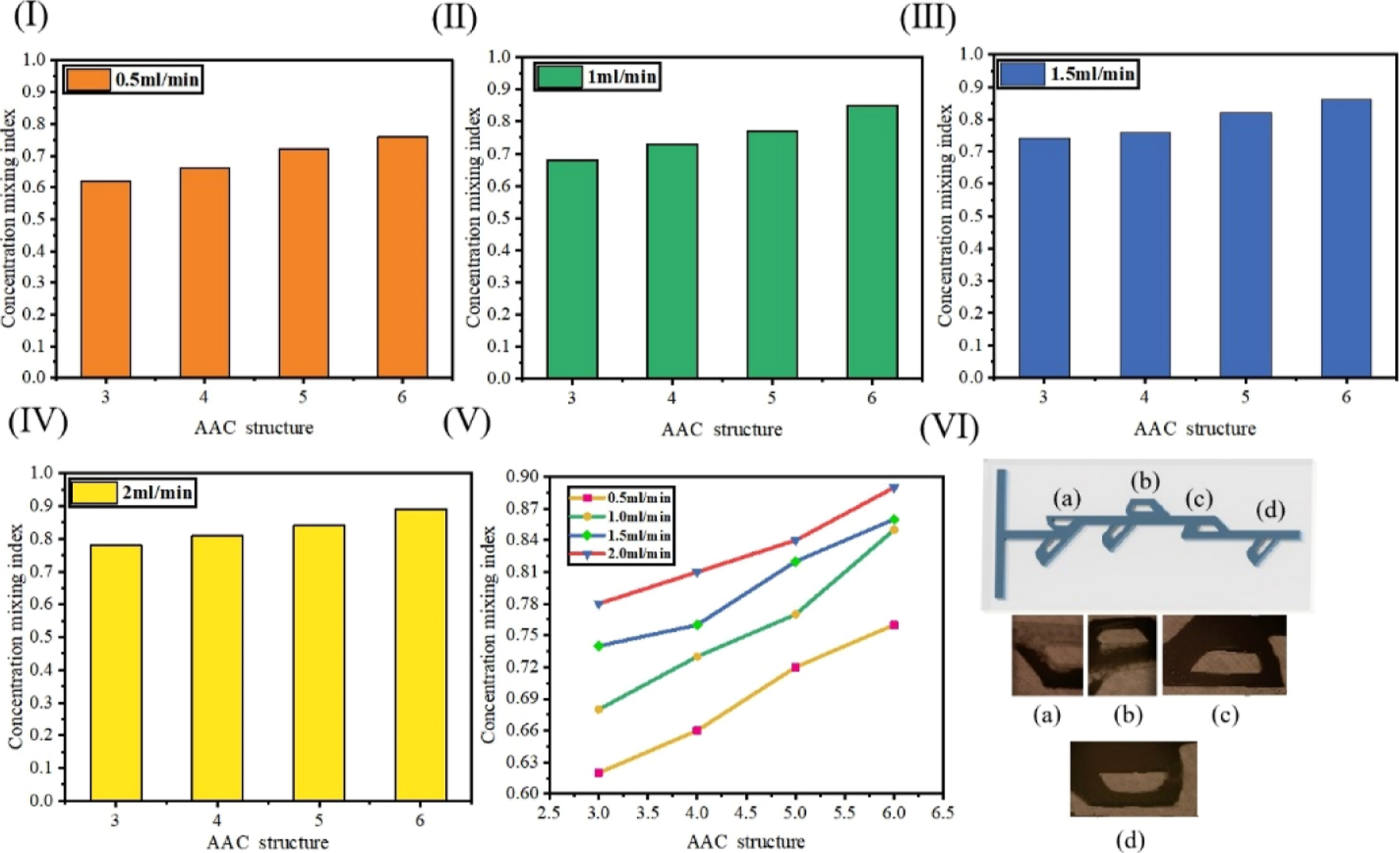
Effect of different flow rates on the mixing efficiency. (I) Micromixer at 0.5 mL/min mixing efficiency. (II) Micromixer at 1 mL/min mixing efficiency. (III) Micromixer at 1.5 mL/min mixing efficiency. (IV) Micromixer at 2 mL/min mixing efficiency. (V) Mixing efficiency of the micromixer at different flow rates. (VI) Mixing effect diagram of the micromixer. (a–d) are the mixing efficiencies of the Tesla valve micromixer at different positions.

Figure 8 shows the mixing efficiency curves of micromixers with the Tesla valve structure under different flow rates with three different groove numbers. Figure 8I–IV shows the micromixer in 0.5, 1, 0.5, and 2 mL/min in different grooves for the Tesla valve structure of the micromixer mixing efficiency. With the increase of flow velocity, the pressure of the fluid in the channel increases, the intermolecular force of the fluid is accelerated, and the mixing efficiency is improved. Figure 8V shows the comparison diagram of the mixing efficiency trend of micromixers with four types of groove Tesla valve structure at four flow rates. Figure 8VI shows the local physical measurement diagram under the microscope. As can be seen from the figure, with the increase of flow rate, the mixing efficiency of the micromixer is improved. Finally, the 6-AAC Tesla valve micromixer can achieve the maximum mixing efficiency of 0.891 at 2.0 mL/min.

### 4.4 Effect of Deformation Conditions

**Figure 9:**
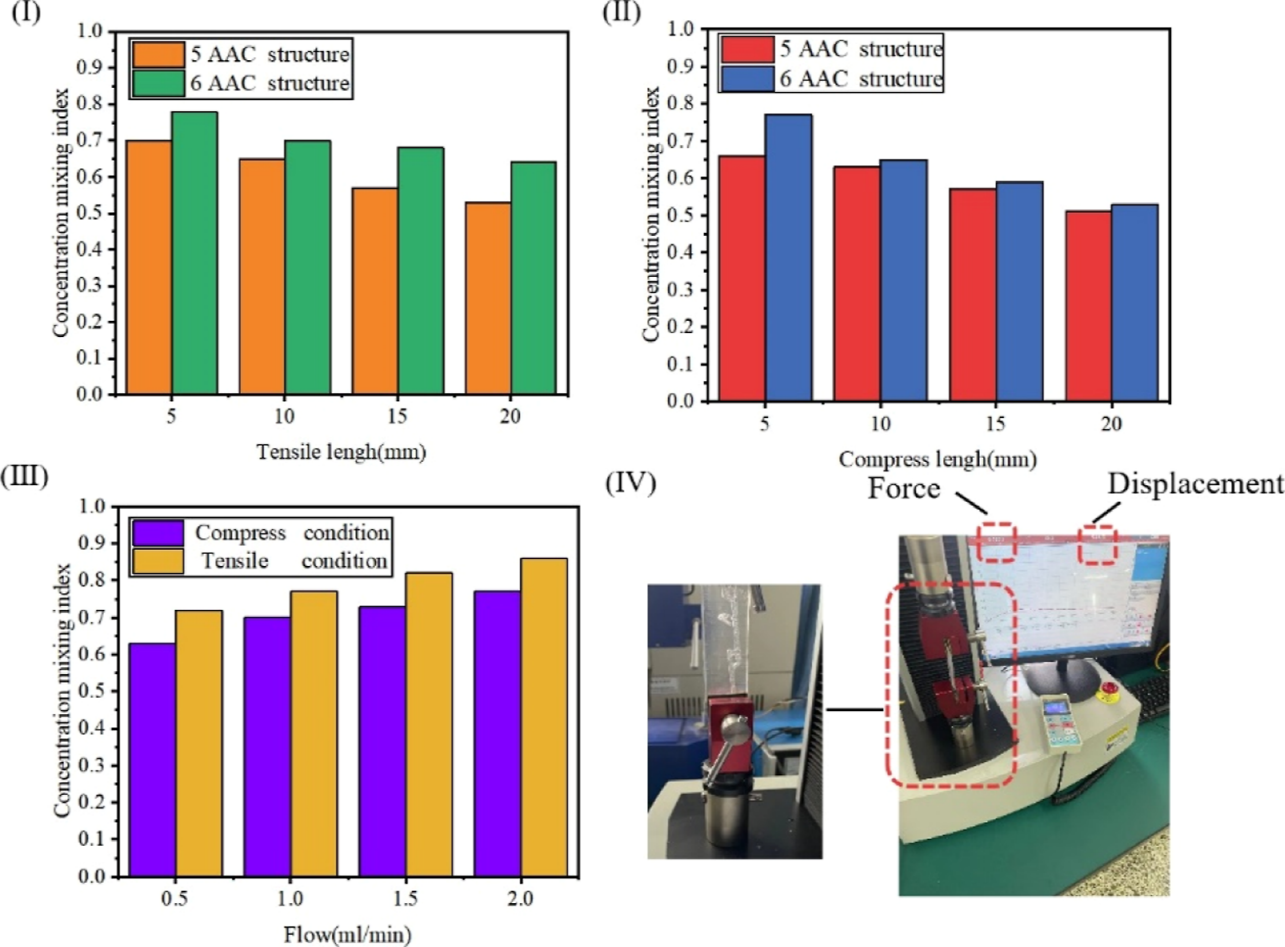
Mixing efficiency of the Tesla valve structure micromixer under different deformation conditions. (I) Mixing efficiency of the micromixer under tensile condition. (II) Mixing efficiency of the micromixer under compression condition. (III) Mixing efficiency of the micromixer under tensile condition and compression condition. (IV) Experimental setup.

Figure 9 illustrates the mixing efficiency of the micromixer with the Tesla valve structure under different deformation conditions. Figure 9I shows the mixing efficiency of the micromixer under tensile condition. With the increase of the tensile length, the mixing efficiency decreases. Since the cross-sectional area of the channel becomes smaller under the stretching condition of the micromixer, the contact area between the fluids becomes smaller, and the flow rate of the microfluidic fluid per unit time decreases. Therefore, the complementary reaction between the microfluidic layers is reduced. At this time, the fluid molecules cannot generate a great slip in a large range, the collision frequency between the molecules is low, and the mixing efficiency is reduced. Figure 9II shows the mixing efficiency of the micromixer under compression condition. When the Tesla valve micromixer is in the compression state, the mixing efficiency will be reduced due to the bending deformation of the microchannel during compression. It is also damaged, resulting in a great change in the microfluidic flow rate and force at the groove, and the collision frequency between the fluid molecules and the solid wall at the groove is low. It can be seen from eq 9 that when the flow rate and width of the microchannel decrease, Pe decreases, the proportion of convection generated between the fluid molecules decreases, and the mixing efficiency decreases. Figure 9III is a comparison of the mixing efficiency between drawing and compressing in a micromixer at a flow rate of 0.5–2 mL/min. Figure 9IV is the experimental setup. Finally, the mixing efficiency of the micromixer is 0.825 when the flow rate is 2 mL/min and the drawing length is 5 mm.
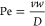
9

Among them, Pe is the relative ratio of convection and diffusion, *v* is the velocity, *w* is the width of the channel, and *D* is the intermolecular diffusion coefficient.

### 4.5 Real Application

**Figure 10:**
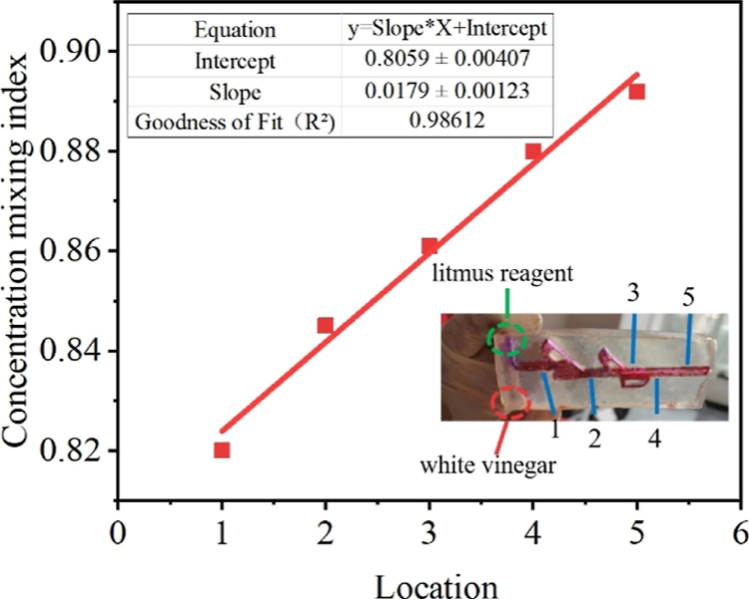
Mixing experiment of the reaction between the purple litmus reagent and white vinegar.

The current micromixer can be widely used in chemical synthesis, cell separation, biopharmaceutical, and other fields. D. J. Kim designed a micromixer similar to crocodile teeth. This micromixer can monitor the ability of glucose–enzyme reaction by adding a triangular structure in a rectangular channel and finally forming a micromixer with a zigzag microchannel. Fluorescence detection was performed by microscopy by introducing glucose and AmplexRed in both inlets. In contrast, the Tesla valve micromixer has smaller size and microchannels and is simple to manufacture, which is more conducive to the mixing of glucose and enzymes and can better complete the spectroscopic experiments of the glucose catalyst reaction. In addition, the Tesla valve micromixer can also test the acidity and alkalinity of chemical reagents, such as purple litmus reagent and white vinegar, colorless phenolphthalein reagent and lime water, and so forth. Figure 10 shows the effect of purple litmus reagent and white vinegar in Tesla. In the mixing experiment under the valve micromixer, it can be seen that the litmus reagent and white vinegar are finally mixed to form a red solution, which proves that white vinegar is acidic. After processing the gray value and calculating the mixing efficiency, it can be seen that with the increase of mixing time, the mixing efficiency gradually increases, and the mixing efficiency is the highest at the time of Location 6, which is 0.893. At the same time, after fitting, *R*^2^ is 0.986, which proves that the mixing efficiency increases linearly.

### 4.6 Comparison of Tables

As the micromixer structure becomes more diverse,^6,15^ this article lists and compares four micromixers, such as Table 6. Although the H-shaped subchannel micromixer is simple to manufacture,^13^ the mixing efficiency is low. Nd3 double layer structure micromixer improves the mixing efficiency by the micro-vortex mixing principle.^12^ However, due to the special microchannel of the double-layer structure, the micromixer needs to be prepared by bonding, and the processing time is too long and the processing is cumbersome.^22^ The three-dimensional fabrication of the spiral channel made by polymer dissolution technology is relatively simple and low cost. Nevertheless, the micromixers do not have unidirectional flow. The peristaltic micromixer has the characteristics of unidirectional flow through the piezoelectric driving method. However, when the fluid inside the micromixer is exposed to the electric field, the transportation mode of the internal fluid is likely to change the chemical properties of the reagent itself, thus affecting the experimental results. In this paper, a new passive micromixer with the Tesla valve structure is proposed, which not only has a simple manufacturing method and is of low cost but also has a high mixing efficiency and unidirectional flow characteristics.

**Table 6:** Comparison of Different Characteristics of Micromixers

microchannel structure	flow mode	mixing efficiency	production	unidirectional liquidity	driving mode
Tesla valve	laminar flow	0.887	30 min	yes	passive
H-shaped subchannels	laminar flow	0.65	2 h	no	passive
spiral channel	turbulence	0.948	30 min	no	passive
Nd3 double layer structure	turbulence	0.94	5 h	no	passive
peristaltic Micromixer	turbulence	0.98	over 24 h	yes	piezoelectric drive

## 5 Conclusions

This paper introduces a micromixer with Tesla valve structure and its preparation method. The mixer has the advantages of one-way flowability and high mixing efficiency.

A: The mixing efficiency of the T-shaped straight channel is 0.7 and that of Tesla valve is 0.865 at the same flow rate compared with that of T-shaped straight channel with the same length and cross-sectional area under different *Re*. Mixing efficiency increased by 0.165. Therefore, the Tesla valve configuration can increase the mixing efficiency compared to the planar configuration. Mixing efficiency increased by 0.165. So, the Tesla Valve configuration can increase the mixing efficiency compared to the planar configuration.

B: The structure of the Tesla valve is characterized by one-way flow. With the increase of the groove structure, the ratio *D*_i_ of the forward pressure to the reverse pressure increases. Finally, the *D*_i_ values of 1.82, 2.5, and 2.9 were obtained when the flow velocity was 6 L/min. In addition, it can be concluded that the increase of the groove structure can increase the unidirectional flowability of the micromixer.

C: Tesla valve construction improves the mixing efficiency. Under the same cross-sectional area, the mixing efficiency of 4-AAC Tesla valve is 0.795 by increasing the groove structure. The mixing efficiency of the 5-groove Tesla valve structure is 0.86. The mixing efficiency of the 6-AAC Tesla valve structure is 0.88. The cross-sectional area of the channel is the same, and the mixing efficiency of the Tesla valve can be improved by increasing the number of grooves.

D: The mixing efficiency of the micromixer with different flow rates is tested under the same structure, and the mixing efficiency increases with the increase of flow rate. The results show that the maximum mixing efficiency is 0.887 under the condition of 2.0 mL/min.

E: It is concluded that the mixing efficiency of the micromixer is reduced under the deformation condition. When the stretch and compression length are the same, the mixing efficiency of the micromixer under the stretch condition is higher than that under the compression condition. The mixing efficiency decreases with the increase of the stretching and compression length. The results show that the mixing efficiency is 0.84 when the flow rate is 2.0 mL/min and the stretch length is 5 mm.

To sum up, the micromixer presented in this paper can effectively improve the mixing efficiency compared with the conventional plane structure. Finally, the 6-AAC Tesla valve micromixer has the best mixing performance when the flow rate is 2.0 mL/min.
